# The Maintenance of Single-Locus Polymorphism by Maternal Selection

**DOI:** 10.1534/g3.115.017392

**Published:** 2015-03-19

**Authors:** Hamish G. Spencer, Kai X. Chiew

**Affiliations:** Allan Wilson Centre, Department of Zoology, University of Otago, Dunedin 9054, New Zealand

**Keywords:** Gavrilets model, multiple alleles, natural selection, mathematical model

## Abstract

Population geneticists have long been interested in the ability of natural selection to maintain the levels of standing variation observed in natural populations. Here, we study the polymorphism-maintaining properties of maternal selection, in which the fitness of an individual is a function of its own and its mother’s genotype. Using a model proposed by Gavrilets, we first estimate the proportion of parameter/state space that preserves allelic variation, before investigating the construction of polymorphism over time through the joint action of mutation and selection. These two methods, the “parameter-space” and “constructionist” approaches, respectively, enable us to draw some general conclusions. We argue that, even though the proportion of parameter-state space allowing multiallele polymorphism is greater under maternal selection than under the standard model of constant viability selection, the former is, in fact, less likely to maintain large numbers of alleles. Nevertheless, variation that is balanced by maternal selection is likely to show elements of heterozygous advantage and be resistant to depletion by genetic drift. We observe that the population mean fitness frequently decreases after the successful invasion of a new mutation, but such declines are usually temporary.

One of the long-standing goals of evolutionary genetics is to understand the relative importance of the various processes that shape genetic variation in natural populations (Lewontin 1974; Kimura 1984; Mitchell-Olds *et al.* 2007; Hahn 2008; Leffler *et al.* 2012; Delph and Kelly 2014; Gao *et al.* 2015). Especially after the advent of gel electrophoresis in the 1970s had revealed large levels of variation in many natural populations, scientists have been interested in the part played by natural selection. Does it actively shape standing variation (as in the balance school; Lewontin 1974), or is its role confined to removing deleterious mutations (as in the neutral hypothesis; Kimura 1984)?

The ascendency of the neutralist view in the 1980s was, in part, due to theoretical problems underlying the balance school. Most critically, Monte-Carlo simulation studies showed that the simplest form of selection, constant viability differences among genotypes, were only able to maintain more than three to four alleles for an extremely restricted set of fitness values (Lewontin *et al.* 1978; Gillespie 1977). These results have been extended to models of several other types of viability selection, such as sexually differential selection (Marks and Ptak 2001), frequency-dependent selection (Trotter and Spencer 2007), and spatially heterogeneous selection (Star *et al.* 2007a), as well as constant fertility selection (Clark and Feldman 1986).

Although such results often were interpreted as showing that selection could not maintain large numbers of alleles (*e.g.*, Árnason 1982; Kimura 1984; Keith *et al.* 1985; Nevo 2001; Charlesworth 2006; Gloria-Soria *et al.* 2012; Ejsmond *et al.* 2014), such a conclusion is logically flawed. Quite generally, the size of some region of parameter space affords little or no information about the likelihood that real parameters lie in that region. Indeed, organic evolution is well recognized to produce systems with exceptionally unusual outcomes (and hence also parameters). In a response to this realization, Spencer and Marks (1988, 1992) and Marks and Spencer (1991) showed that a simple system combining recurrent mutation and natural selection iterated toward those parts of viability-parameter space in which numerous alleles could be maintained. Polymorphism was naturally “constructed.” This result, using the so-called “constructionist approach,” holds for numerous models of selection and mutation (Marks and Ptak 2001; Star *et al.* 2007b, 2008; Trotter and Spencer 2008, 2013). We note that this approach has significant commonalities with the later approach of adaptive dynamics (Waxman and Gavrilets 2005; but see also Spencer and Feldman 2005).

In this paper, we investigate the ability of maternal selection to maintain genetic variation. Many organisms’ phenotypes are profoundly affected by their maternal environment (Mousseau and Fox 1998; Mousseau 2006; Wolf and Cheverud 2012), but population-genetic models of the selective consequences of these effects have been developed only in the last 20 years (Gavrilets 1998; Wade 1998; Spencer 2003; Spencer *et al.* 2006). In our investigations we first determine the size of parameter/state space that leads to polymorphism under Gavrilets’s (1998) model of maternal selection, before using the constructionist approach to assess the likelihood of polymorphism under this model.

## Model

We start by generalizing the 2-allele model of maternal selection of Gavrilets (1998; see also Spencer 2003). Consider a single locus with *n*alleles, *A*_1_, *A*_2_, …, *A_n_*, at respective frequencies *p*_1_, *p*_2_, …, *p_n_* (with $\sum_{i=1}^{n}p_{i}=1$), in a randomly mating, dioecious population, in which the effects of mutation and genetic drift are negligible.

To model maternal selection, we must parameterize each combination of offspring-mother genotypes (Cheverud and Wolf 2009; Wolf and Wade 2009; Wolf and Cheverud 2012), say an *A_i_A_j_* offspring with an *A_k_A_l_* mother, where *i* = *k* or *l*, or *j* = *k* or *l*, or both) by a fitness parameter, *w_ijkl_*. As in most models of selection, only the relative size of these fitnesses matters, and so we assume that 0 ≤ *w_ijkl_* ≤ 1. It is critical to ensure the various combinations are correctly enumerated. For *n* alleles, there are *n* possible homozygotes and $\left(\begin{array}{c} n\\ 2 \end{array}\right)$ different heterozygotes. Each homozygote, say *A_i_A_i_*, can have *n* possible different mothers, *A_i_A*_1_, *A_i_A*_2_, …, *A_i_A_n_*, which makes for *n*^2^ combinations. Each heterozygote, say *A_i_A_j_* (*i* ≠ *j*), can have two sorts of homozygous mothers, *A_i_A_i_* and *A_j_A_j_*, one identical (heterozygous) mother, *A_i_A_j_*, and 2(*n* – 2) nonidentical heterozygous mothers, *A_i_A_k_* and *A_j_A_k_* (where *k* ≠ *i*, *j* can take on *n* – 2 different values). Hence, the number of heterozygote-offspring and mother combinations is

$$\left(\begin{array}{c} n\\ 2 \end{array}\right)\left(2+1+2\left(n-2\right)\right)=\frac{1}{2}n\left(n-1\right)\left(2n-1\right)$$ (1)

and so, the number of distinct fitness parameters is $C\left(n\right)=n^{2}+\frac{1}{2}n\left(n-1\right)\left(2n-1\right)$. These numbers rapidly become large: for *n* = 1, 2, 3, 4, and 5, we require, respectively, 1, 7, 24, 58, and 115 distinct fitness parameters.

Accordingly, there are five distinct classes of offspring-mother combinations: homozygotes with homozygous mothers, homozygotes with heterozygous mothers, heterozygotes with homozygous mothers, heterozygotes with identical heterozygous mothers, and heterozygotes with nonidentical heterozygous mothers. The relative sizes of the fitnesses of these different classes might be expected to be differentially important in maintaining variation. For a given fitness set, we denote the mean of the fitnesses in each class by, respectively, *C_iiii_*, *C_iiij_*, *C_ijii_*, *C_ijij_*, and *C_ijik_*. Note that for smaller values of *n* some of these classes are empty.

Maternal selection requires that we keep track of genotype frequencies. Let *x_ij_* (= *x_ji_*) be the frequency of genotype *A_i_A_j_* (so that $\sum_{i=1}^{n}\left(x_{ii}+\sum_{j=i+1}^{n}x_{ij}\right)=1$) and so $p_{i}=x_{ii}+\frac{1}{2}\sum_{j\neq i}^{n}x_{ij}$. *A_i_A_i_* homozygotes always receive an *A_i_* allele from their father with frequency *p_i_*. They also inherit an *A_i_* allele from their mother, either an *A_i_A_i_* homozygote, with frequency *x_ii_*, or an *A_i_A_k_* heterozygote (for any *k* ≠ *i*), with frequency 1/2*x_ik_*. These two types of mothers confer different fitnesses on their offspring, *w_iiii_* and *w_iiik_*, respectively. Hence, the iteration for the homozygote frequencies is given by

$$\bar{w}{x^{\prime }}_{ii}=p_{i}\left(w_{iiii}x_{ii}+\frac{1}{2}\sum_{k\neq i}^{n}w_{iiik}x_{ik}\right)$$. (2)

An *A_i_A_j_* heterozygote can receive its *A_i_* allele from its father and its *A_j_* from its mother (who, as mentioned previously, may be homozygous or heterozygous for the *A_j_* allele), or vice versa, giving rise to the two terms on the right-hand side in the following equation. Using the same logic as described previously, we find that the iteration for heterozygote frequencies is given by

$$\bar{w}{x^{\prime }}_{ij}=p_{i}\left(w_{ijjj}x_{jj}+\frac{1}{2}\sum_{k\neq j}^{n}w_{ijjk}x_{jk}\right)+p_{j}\left(w_{ijii}x_{ii}+\frac{1}{2}\sum_{k\neq i}^{n}w_{ijik}x_{ik}\right)$$, (3)

for *i* ≠ *j*. In these equations, $\bar{w}$ is the mean fitness, the sum of the right-hand sides of Equations (2) and (3) for *i* = 1, 2, 3, …, *n* and *j* > *i*. This formulation ensures that the genotype frequencies in the following generation also sum to one: $\sum_{i=1}^{n}\left({x^{\prime }}_{ii}+\sum_{j=i+1}^{n}{x^{\prime }}_{ij}\right)=1$.

### Estimating potential for polymorphism

For a fixed number of alleles, *n* = 2, 3, …, 6, we estimated the proportion of viability-parameter space and initial genotype-frequency state-space that maintained all *n* alleles. This proportion we call the “potential” for polymorphism and the method the “parameter-space approach,” following Trotter and Spencer (2007). We did so by first generating 10^5^ random sets of *C*(*n*) fitnesses. As in Lewontin *et al.* (1978), these fitnesses were drawn from the uniform distribution between zero and one, U[0, 1]. We do not wish to imply that natural fitnesses are uniformly distributed; rather, the use of U[0, 1] allows us to model parameter space as a *C*(*n*)-dimensional cube, which provides for a robust method for estimating the potential for polymorphism (see also Trotter and Spencer 2007).

Under constant viability selection, a given fitness set has at most one fully polymorphic equilibrium (Bürger 2000), but such a property does not hold under maternal selection (Gavrilets 1998; Spencer 2003), and, indeed, allele-frequency cycling is possible but rare (Spencer 2003). In other words, iteration of Equations (2) and (3) from different (nontrivial) initial genotype frequencies does not necessarily lead to a unique genotype-frequency vector even if all *n* alleles are retained. Rather, for each fitness set, we determined the fraction of initial genotype frequencies that lead to an *n*-allele polymorphism (either an equilibrium or the cycling of *n* alleles). Thus, for each fitness set, we iterated from 100 randomly selected initial genotype frequencies and recorded the proportion that retained *n* alleles. If all 100 initial genotype frequencies for a particular fitness set retained all *n* alleles, we called this fitness set an “always” fitness set; if only some iterations retained all *n* alleles, the fitness set was described as a “sometimes” fitness set. The initial genotype frequencies were derived from randomly generated allele frequencies found using the broken-stick method (Holst 1980) and assuming Hardy-Weinberg genotype proportions.

The potential for polymorphism for a given *n* is thus given by the proportion of initial genotype-frequency vectors over all 10^5^ fitness sets that iterated to an *n*-allele equilibrium or cycled with *n* alleles. This value is equal to the proportion of “always” fitness sets plus the proportion of iterations over all “sometimes” fitness sets that maintained *n* alleles.

### Constructing polymorphisms

Following the approach outlined in Spencer and Marks (1988), we began our constructionist simulations with a monomorphism (*n* = 1) and assumed *w*_1111_ = 0.5. Every generation, a new mutant allele, *A_n_*_+1_ was added to the existing *n*-allele system at a frequency of *p_n_*_+1_ = 10^−5^ and the frequency of one randomly chosen allele already present in the population was reduced by the same amount. The addition of (*n* + 1)^th^ allele results in *n* + 1 new genotypes and hence $C\left(n+1\right)-C\left(n\right)=3n^{2}+2n+1$ new random fitnesses must be generated. These parameter values were drawn from U[0, 1]. Again, we do not wish to imply that fitnesses in natural populations have such a distribution, but doing so provides a more straightforward comparison with the results of other forms of selection.

We then iterated genotype (and hence allele) frequencies according to Equations (2) and (3). Any allele whose frequency fell below 10^−5^ was considered to be extinct and was removed from the system. Each generation we recorded the number and frequencies of all alleles, as well as the mean fitness. After 10^4^ generations each simulation was stopped and the fitness set of the extant alleles recorded.

We ran 10^3^ replicate simulations, differing only in the seed for the pseudo-random number generator. In all our simulations we used the pseudo-random number generator of Marsaglia *et al.* (1990). The programs used in our simulations may be found in Supporting Information, File S1 and File S2.

## Results

### Potential for polymorphism

**Figure 1:**
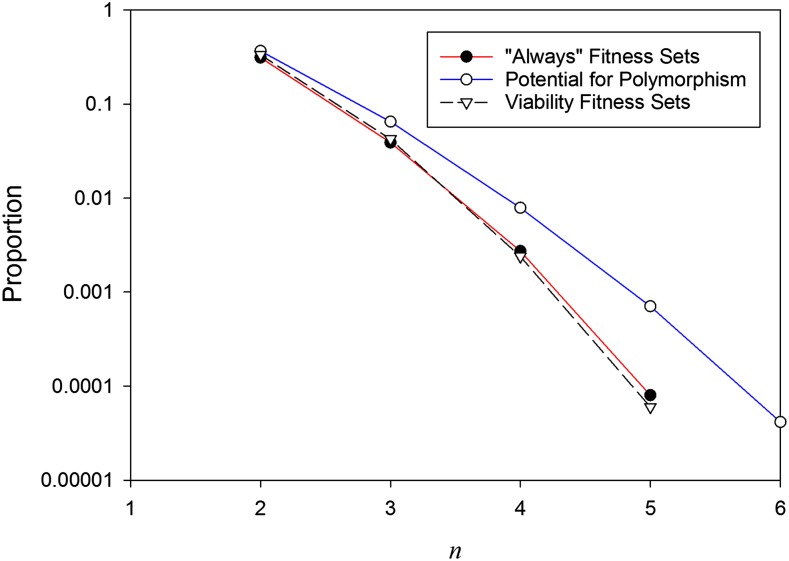
Parameter/state space maintaining all *n* alleles. The blue line with open circles shows the potential for polymorphism under maternal selection, *i.e.*, proportion of parameter/state space for which all *n* alleles are maintained for random fitness sets and initial genotype frequencies. The red line with filled circles shows the proportion of maternal fitness sets that always maintained all *n* alleles, *i.e.*, the proportion of “always” fitness sets. The corresponding values for constant viability selection (Lewontin *et al.* 1978) are shown in dashed black.

The proportion of parameter/state space that affords full (*n*-allele) polymorphism is shown in Figure 1. Also plotted is the proportion of “always” fitness sets, and the corresponding results for constant viability selection from Lewontin *et al.* (1978) are shown for comparison. [Under constant viability selection, when an *n*-allele equilibrium is possible, all (nontrivial) initial genotype frequencies iterate to this equilibrium, and so there is just a single line.] Table 1 also shows the numbers of “always” and “sometimes” fitness sets for each value of *n*. The proportion of initial genotype frequencies that maintained all *n* alleles for the latter type of fitness set declined with *n* (Table 1).

**Table 1:** Results from “parameter-space” simulations

*n*	No. of “Always” Fitness Sets (out of 100,000)	No. of “Sometimes” Fitness Sets (out of 100,000)	Proportion of Iterations Maintaining *n* Alleles for “Sometimes” Fitness Sets	Potential for Polymorphism
2	31,154	7842	0.7068	0.36697
3	3894	3825	0.6814	0.06500
4	273	891	0.5769	0.00787
5	8	134	0.4663	0.00070
6	0	13	0.3192	0.00004

Clearly, the potential for polymorphism declines with *n*, a result that holds for all forms of selection investigated to date. The potential for polymorphism under maternal selection is, however, greater than that under constant viability selection, whereas the proportion of fitness sets that always maintain all alleles is very similar for the two forms of selection.

Allele-frequency cycling was very rare, with 0, 65, 44, 12 and one cases for *n* = 2, 3, 4, 5 and 6, respectively, of the 10^5^ fitness sets for each value of *n*.

### Constructing polymorphisms

**Figure 2:**
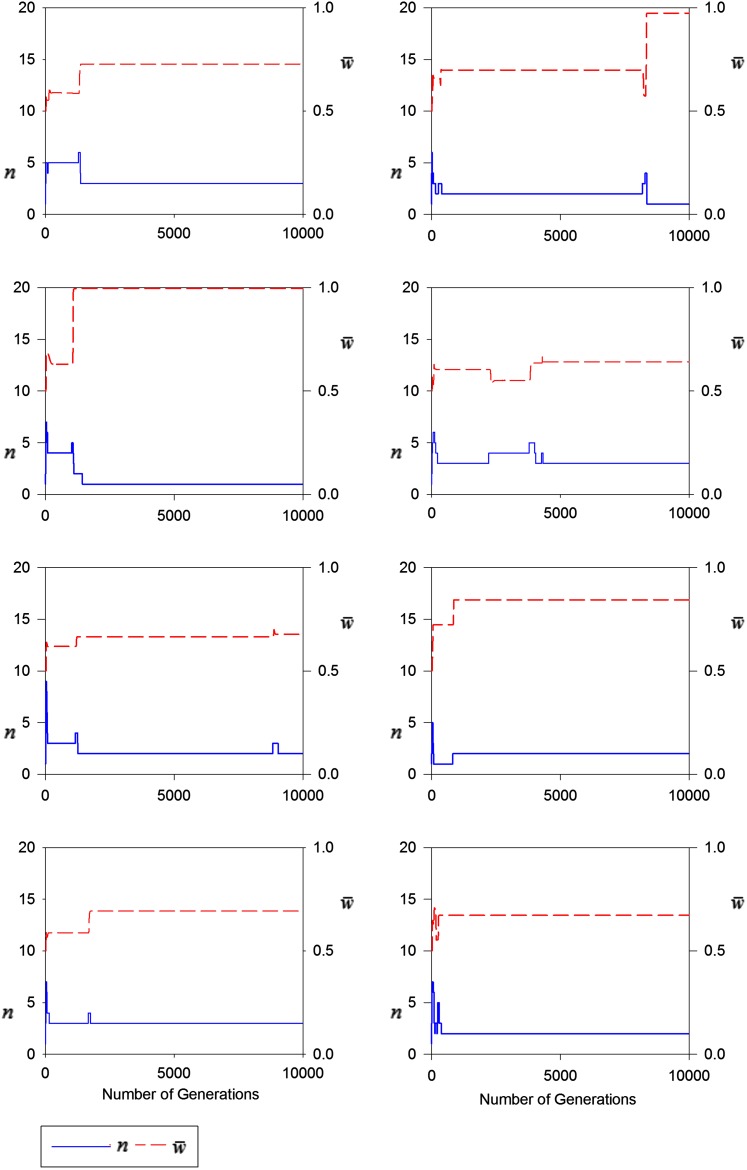
Representative simulations of the construction of polymorphism maintained by maternal selection. Upper (dashed red line) shows the mean fitness of the population, $\bar{w}$, and the lower (solid blue) line shows the numbers of alleles over Generations 0−10,000.

The results of some representative constructionist simulations are shown in Figure 2. In each simulation, the number of alleles rapidly increases, but soon drops to ≤ 5. Long periods of no change in this number mean that no new mutations have successfully invaded. In several cases, successful invasion is quickly followed by the extinction of one or more alleles; the successful mutation is driving out some of the standing variation. The mean fitness generally increases, but this increase is not monotonic. Significant changes (especially increases) in the mean fitness coincide with changes the numbers of alleles (*i.e.*, successful invasions and extinctions).

**Figure 3:**
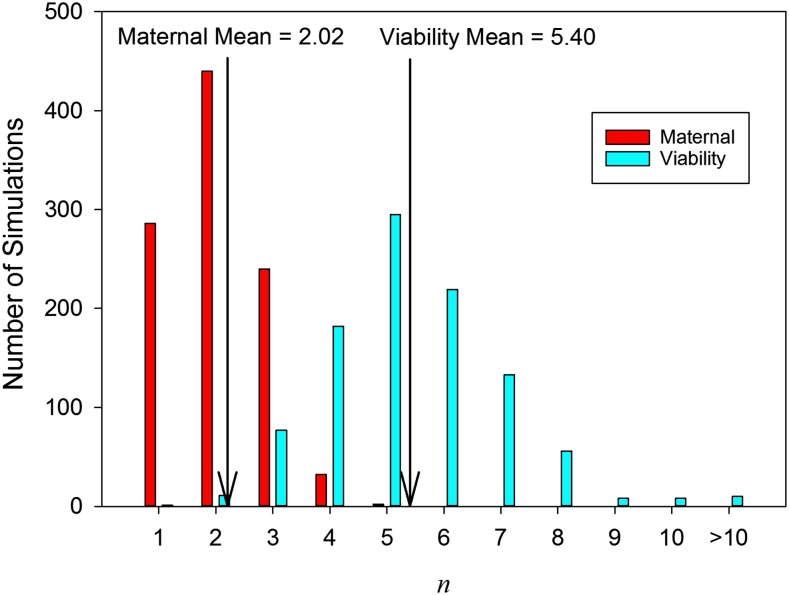
Bar chart of red bars showing the number of alleles at Generation 10^4^ in 1000 replicate simulations of the constructionist simulations of maternal selection. Also shown, for the sake of comparison, is the distribution for constant viability selection using data from Spencer and Marks (1992) (blue bars).

The distribution of the number of alleles in each of the 1000 simulations after 10^4^ generations is shown in Figure 3. Also shown is the distribution under constant viability selection (data from Spencer and Marks 1992). The modal number of alleles under maternal selection is just 2 (with a mean of 2.04), significantly lower than the modal number of 5 (and mean of 5.4) under viability selection. Thus, the larger potential for polymorphism under maternal selection (as compared to constant viability selection) is not realized in a larger number of alleles in constructed polymorphisms.

**Figure 4:**
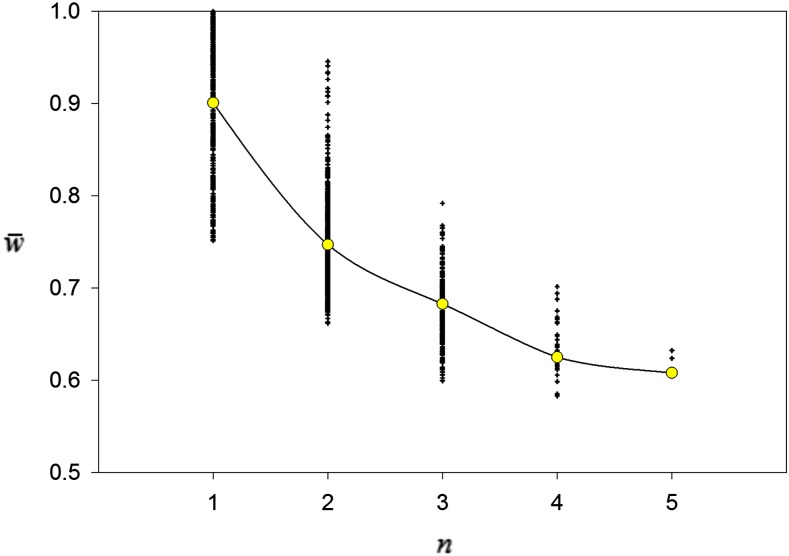
Relationship between the number of alleles (*n*) and population mean fitness ($\bar{w}$) at Generation 10^4^ in 1000 replicate simulations of the constructionist simulations of maternal selection. Mean values plotted in yellow circles.

Constructed polymorphisms with larger numbers of alleles tended to have lower mean fitnesses (Figure 4). Successful invasion by a mutant allele is more likely, everything else being equal, if the mean fitness is lower, and so polymorphisms with lower mean fitnesses are likely to be invaded more easily, leading to greater numbers of alleles. Such dynamics also could explain why polymorphisms with a large number of alleles are rare: easy invasion may soon be followed by one or more extinctions, lowering the numbers of alleles.

**Figure 5:**
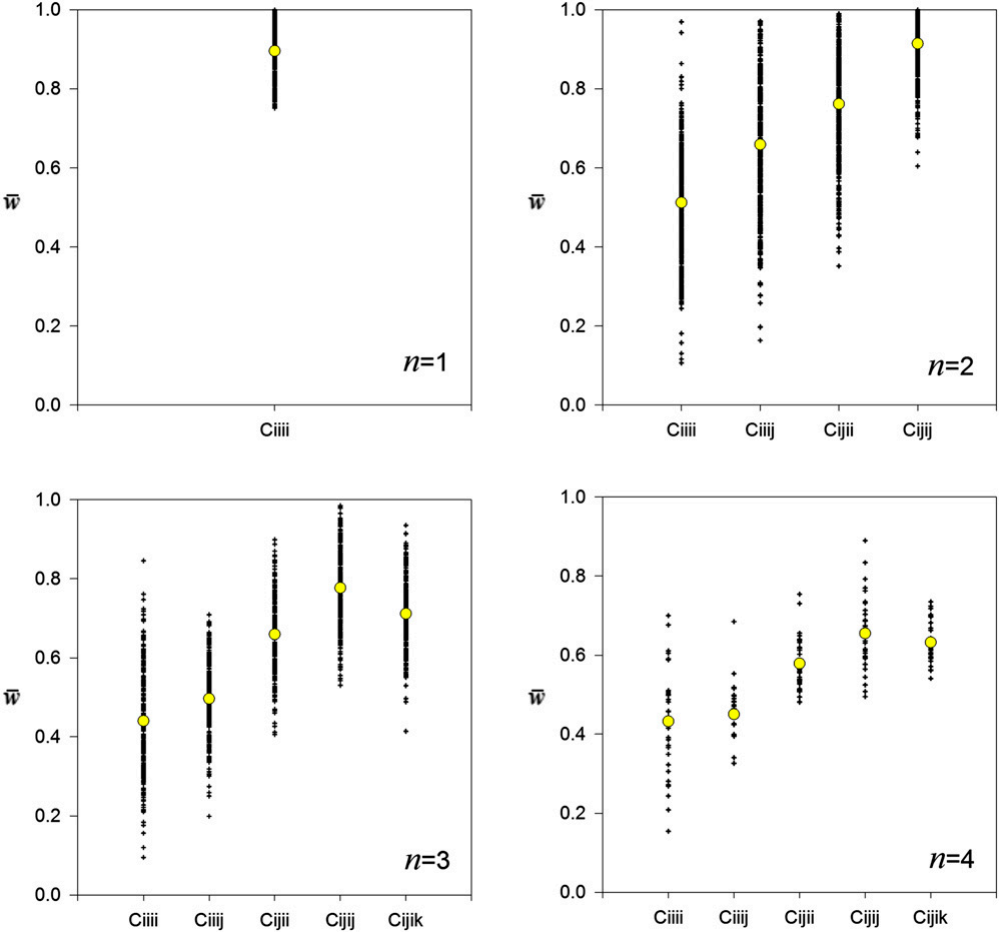
Mean fitnesses for different classes of offspring-mother combinations at Generation 10^4^ in 1000 replicate constructionist simulations of maternal selection, plotted for different final numbers of alleles (*n*). Combination *C_iiii_* is the mean of homozygous offspring with homozygous mothers, for example, whereas *C_ijij_* is the mean of heterozygous offspring with identically heterozygous mothers. For a full explanation see text. Means for each class are shown in yellow circles.

Figure 5 shows the mean fitnesses in each simulation for the different classes of offspring-mother combinations for each value of *n* for which there were at least 10 simulations (*i.e.*, 1 ≤ *n* ≤ 4). The pattern is consistent: heterozygous offspring tended to have higher fitnesses than homozygous offspring, differences that were accentuated if their mothers were also heterozygous (*i.e.*, *C_iiii_* < *C_iiij_* < *C_ijii_* < *C_ijij_* and *C_ijik_*).

**Figure 6:**
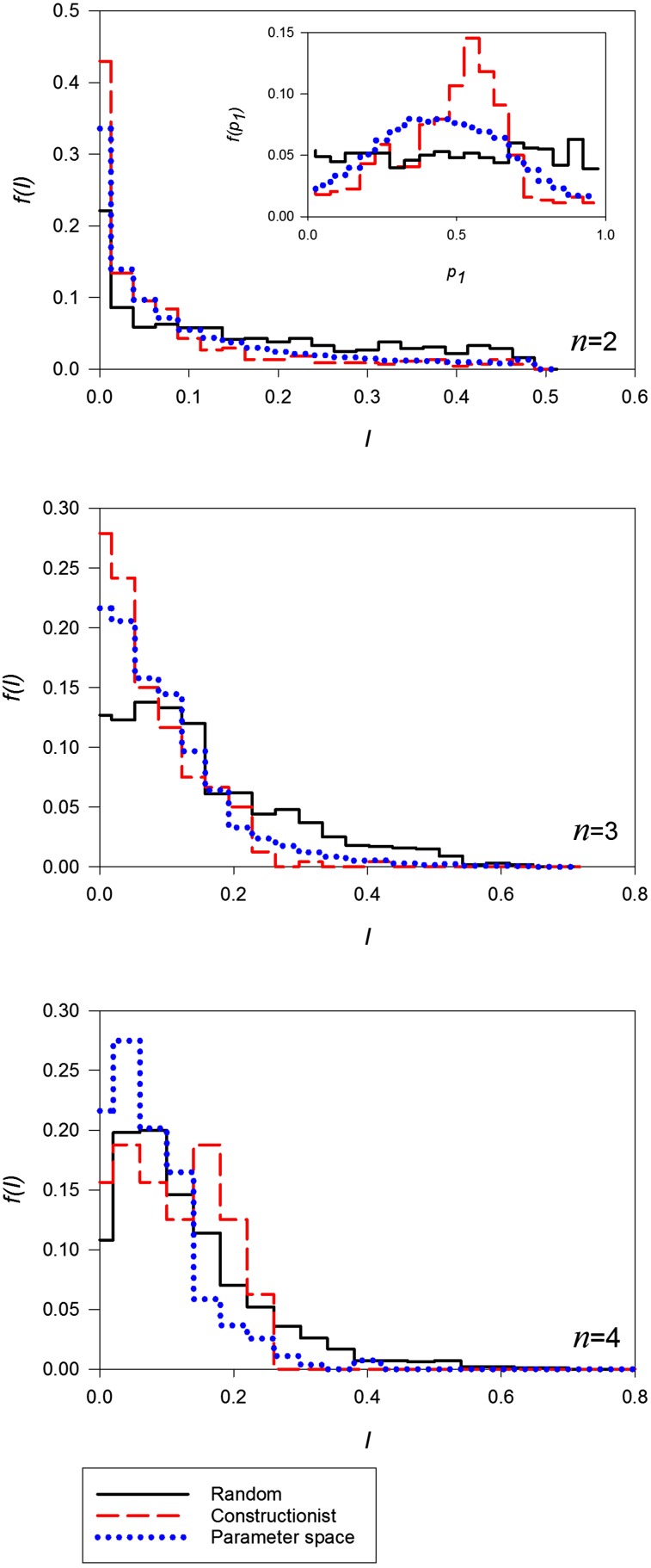
Histograms of $I=\sum_{i=1}^{n}{\left(p_{i}-\frac{1}{n}\right)}^{2}$, a measure of centrality of allele frequencies at equilibria in the parameter-space simulations (blue dotted lines) and at Generation 10^4^ in the constructionist approach (red dashed lines). Also shown (black solid lines) is the distribution expected if allele frequencies were random, values generated using the broken-stick approach. For *n* = 2, the same information is shown as a small histogram of the respective values of *p*_1_.

It is also of interest to know about the values of the allele frequencies at Generation 10^4^: Are these even or skewed? We can summarize information about the distribution using the sum of the squared distances from the centroid of the state space of allele frequencies, $I=\sum_{i=1}^{n}{\left(p_{i}-\frac{1}{n}\right)}^{2}$. Large values of *I* indicate uneven allele frequencies; equal allele frequencies give *I* = 0. The distribution of the values of *I* for *n* = 2, 3, and 4 is shown in Figure 6, along with the values expected in allele frequencies drawn at random under the broken-stick model (Holst 1980). For *n* = 2 we also plot the values of *p*_1_. It is clear that for *n* = 2 and 3 (but not 4), constructed polymorphisms have allele frequencies that are more even than our random expectation. Also shown in Figure 6 are histograms of the values of *I* under the parameter-space approach. For *n* = 2 and 3, these values are intermediate; for *n* = 4, they are more even than both the random and constructed frequencies.

## Discussion

Our investigation of fitness-parameter space under maternal selection suggests that this important form of selection has a greater potential to maintain large numbers of alleles than does standard viability selection. Somewhat surprisingly, however, we also show that this potential is not realized when selection and mutation construct polymorphisms over time: maternal-selection simulations maintain fewer alleles than do those of constant viability selection. This pair of results reinforces the logical point that the size of some portion of parameter space is not informative about the likelihood of a system to lie in that portion.

The difference between viability and maternal selection in their potential to maintain variation is almost entirely caused by the existence of offspring-mother fitness sets that cannot maintain all *n* alleles starting from all (nontrivial) allele frequencies (the “sometimes” fitness sets; Figure 1; Table 1). Indeed, for *n* = 4 and 5, the majority of fitness sets that maintained all *n* alleles did so from only some initial allele frequencies. For such fitness sets there must exist one (or more) allele(s) that, when rare, will be eliminated but, when common, will iterate to a polymorphic equilibrium. If such a fitness set were generated in a constructionist simulation and that same allele were the new mutant, there would be no increase in the number of alleles. Thus, this fitness set would count toward the ability of maternal selection to maintain greater levels of variation under the parameter-space approach than under the constructionist approach. This difference may explain the lower numbers of alleles found in our constructionist simulations.

Under both viability and maternal selection, the conditions for successful invasion of a new mutation become very restrictive as mean fitness increases and fewer alleles can invade (Figure 2; see also Figure 2 in Spencer and Marks 1988). Nevertheless, this constraint is greater in the parameter-rich model of maternal selection because in this model, on average, more fitnesses must be large. Thus, under maternal selection, fewer successful invasions occur and the consequent number of alleles is lower. The maximum number of alleles we observed in our constructionist investigation was 5, which happened only twice in 1000 simulations. A negative correlation between the relative potential for polymorphism (compared to standard viability selection) and its realization have previously been found in investigations of sex-dependent viabilities (Marks and Ptak 2001).

Gavrilets (1998) and Spencer (2003) showed that, under maternal selection, the mean fitness of a population may sometimes decline. Most of our selected simulations in Figure 2 exhibit such decreases, although they are short-lived and, consequently, over long periods of time mean fitness generally increases. Thus maternal selection, like constant viability selection, is likely to lead to improved adaptation.

Perhaps not surprisingly, mean fitness changes most often after the invasion of a new mutation, but this change need not be an increase. Nevertheless, a successful invasion often is followed by the extinction of one or more alleles from the standing variation, accompanied by significant increases in mean fitness. In only a minority of our Figure 2 simulations does a decrease in fitness persist for more than a few generations.

The sorts of fitness sets that maintain polymorphism under maternal selection tend to show a generalized heterozygote advantage (Figure 5). The class of offspring-mother combinations that had the highest mean fitness in our constructionist simulations was that of heterozygous offspring with identically heterozygous mothers (*C_ijij_*). Interestingly, this mean was higher than that of heterozygotes with non-identical heterozygous mothers, with whom they shared just one allele. The lowest class fitness was that of homozygotes with (identical) homozygous mothers (*C_iiii_*).

The relatively even allele frequencies found using both parameter-space and constructionist approaches (Figure 6) is in contrast to that under constant viability selection (Spencer and Marks 1988). In finite populations, genetic drift is likely to reduce the number of alleles in the standing variation (Star and Spencer 2013). Everything else being equal, even equilibrium allele frequencies will be more resistant to extinction due to genetic drift and so maternal selection may play a more important role in maintaining variation in smaller populations than our results might, at first, suggest.
